# Dataset for the morphological and erythrocytes parameters of *Clarias gariepinus, Pangasianodon hypophthalmus,* and their reciprocal hybrids

**DOI:** 10.1016/j.dib.2020.106151

**Published:** 2020-08-07

**Authors:** Victor Tosin Okomoda, Ivan Chu Chong Koh, Anuar Hassan, Abraham Sunday Oladimeji, Mhd Ikhwanuddin, Ambok Bolong Abol-Munafi, Korede Isaiah Alabi, Sheriff Md Shahreza

**Affiliations:** a Department of Fisheries and Aquaculture, College of Forestry and Fisheries, University of Agriculture, Makurdi P M B. 2373, Nigeria; b Institute of Tropical Aquaculture and Fisheries Research (AQUATROP), Universiti Malaysia Terengganu, Kuala Nerus, Terengganu 21030, Malaysia; c Faculty of Food Science and Fisheries, Universiti Malaysia Terengganu, Kuala Nerus, Terengganu 21030, Malaysia; d Agricultural Department, National Biotechnology Development Agency (NABDA), Abuja, Nigeria; e Department of Agricultural Extension and Management, Federal College of Forestry, Jos. Plateau, Nigeria

**Keywords:** African catfish, Asian catfish, Blood smear, Truss network, Exclusive ranges, Hybridization

## Abstract

Discrimination of different fishes can be done through different means which includes morphological appearance. When two fishes are successfully hybridized, they produce progenies that have shared morphology between their pure parent, hence, making morphometric characterization an important aspect of hybrid discrimination. However, erythrocyte characterization is also a simpler method for characterization. The dataset presented in this article represents the traditional morphological data, truss network data and erythrocyte data of pure and novel hybrids from reciprocal crosses of African catfish *Clarias gariepinus* and Asian catfish *Pangasianodon hypophthalmus.* Breeding of the broodstocks was done to produce pure and hybrid progenies which were maintained for a period of four to six months. Based on the cross combinations and morphotypes, traditional measurement of twenty-five morphological characters and five meristic counts were recorded. Thereafter pictures of the different fish groups were used to determine values of thirty-six distances between ten landmark points. The morphological abnormality of the hybrids at market size is also presented in this data article for the very first time. Blood was then collected from the caudal peduncle of ten fish per group and smeared on a slide for observation under a compound microscope (at 100 × magnification). Data gotten included erythrocytes parameters such as cell major axis, cell minor axis, nucleus major axis, nucleus minor axis cell area, nucleus area, cell volume, and nucleus volume. Data recording was through the Microsoft excel spreadsheet; which was also used to process the data to get the exclusive ranges of values for paired progenies. The data as presented is associated with the research article “Morphological characterization of the progenies of pure and reciprocal crosses of *Pangasianodon hypophthalmus* (Sauvage, 1878) and *Clarias gariepinus* (Burchell, 1822)” [1]. The dataset presented in this article can be used for easy identification of the novel hybrid progenies of the African Catfish and Asian Catfish.

**Specifications Table**

**Table:** 

**Subject**	Biological Science
**Specific subject area**	Aquatic Science
**Type of data**	Figures, Tables, and Images
**How data were acquired**	Weighing balance and meter rule for taking weight and length measurement respectively. Photographic pictures were taken with the aid of a Nikon professional camera. Microscopic examination and measurement of the erythrocyte using the Nikon Eclipse 80i compound microscope. Microsoft Excel software for computation of relevant variables displayed in tables.
**Data format**	Raw and Analyzed
**Parameters for data collection**	Fishes whose morphological features were taken were devoid of abnormalities that are considered of genetic origin. Pictorial capture of fish was at an axis in a near-perfect 180° so all the features of the fish can be seen and captured. Erythrocyte measured were normal erythrocytes without abnormality. Measurement was done from one end to another following the axis in a near-perfect 180° angular division of the cell cross-section.
**Description of data collection**	Conventional traditional methods of determining morphometric measurement and meristic counts were done according to the method earlier described by Teugels et al. [2] and Solomon et al. [3]. However, truss network data were measured for the morphological traits in line with the methods of Turan et al. [4] with some modifications based on the peculiarities of the species experimented upon. Erythrocyte slides were observed under the Nikon eclipse 80i compound microscope (at × 100 magnification). The erythrocyte seen on the slide was sectioned into five different blocks out of which ten erythrocytes were measured in each block (*n* = 50 for each slide) following the method described by Normala et al., [5]. The measurement done was cell and nuclear axis (both major and minor). Microsoft Excel software was used to determine the volume, area, excusive ranges and percentages by calculation and sorting of values in ascending order where necessary. Exclusive ranges were also obtained for the morphological dataset.
**Data source location**	Institution: Universiti Malaysia Terengganu City/Town/Region: Terengganu Country: Malaysia
**Data accessibility**	With the article
**Related research article**	V.T., Okomoda, I.C.C., Koh, A., Hassan, T., Amornsakun, M.S., Shahreza, 2018. Morphological characterization of the progenies of pure and reciprocal crosses of *Pangasianodon hypophthalmus* (Sauvage, 1878) and *Clarias gariepinus* (Burchell, 1822). Scientific Reports. 8: 3827 https://doi.org/10.1038/s41598–018–22149–4[1]

**Value of the Data**

• Morphological and erythrocyte characteristics of the novel hybrids between Clarias gariepinus, Pangasianodon hypophthalmus were presented for the first time.
• Data can be used by fish breeders, pathologist, microbiologist and other scientists working on fish identification or hybridization between different Catfishes or hematological studie.
• The morphological data presented is the simplest method for accurately discriminating the novel hybrids by merely looking at some of their specific features.
• The data is a good reference for future studies and comparison with the hybrids of other closely related species of the African catfish and the Asian catfish.
• The exclusive size range of the data determined is a very important index to help prevent invasive species in the natural environment.
• The analysis of the data as given in the Microsoft excel can serve as a guide for future studies in, eliminating size effect from morphological data and developing exclusive ranges of for established and novel hybrids of other fish species using both morphological and erythrocyte parameters.

## 1 Data description

**Fig. 1:**
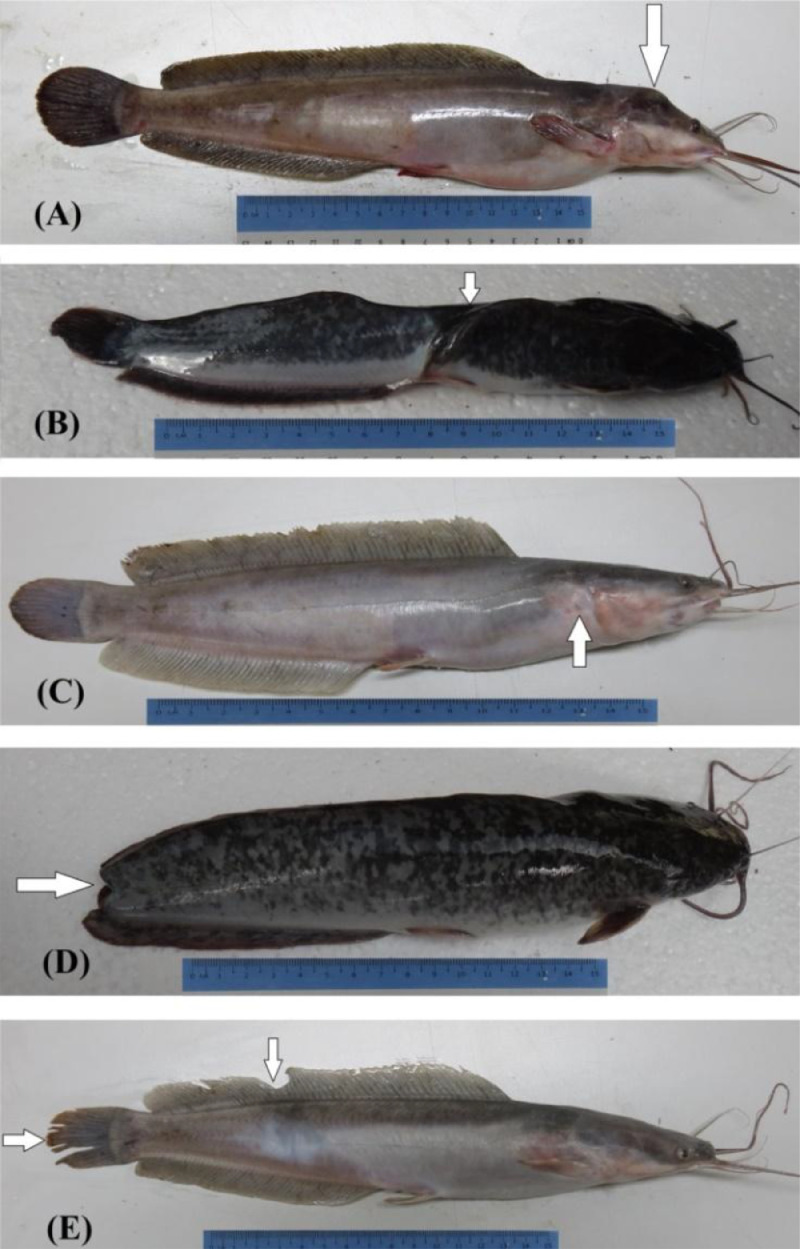
Abnormal Clarias-like Progenies in the crosses of pure and reciprocal hybrids of *Pangasianodon hypophthalmus* and *Clarias gariepinus*. (A) dump head shape (B) abnormal linkage of dorsal and anal fin (C) Absence of pectoral fin (D) cannibalized caudal fin (E) lateral divisions of the dorsal and caudal fins.

**Fig. 2:**
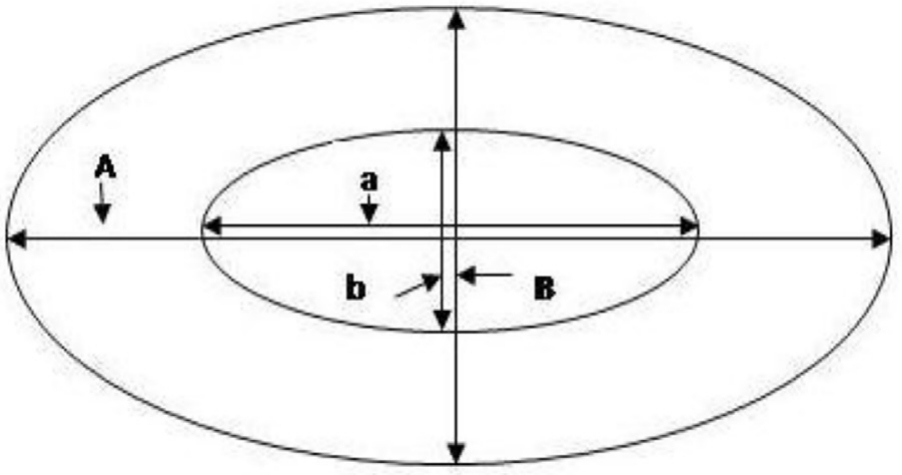
Characteristics of the erythrocyte measured; A (Cell major axis), B (Cell minor axis), a (Nucleus major axis) and b (Nucleus minor axis). Sketch adapted from Normala et al. [5].

The shared raw data encompasses the morphology of fishes gotten through traditional measurement of morphometric parameters, counting of meristic characters (Microsoft excel file name: Analysis traditional method), landmark distances between landmark points of truss networks (Microsoft excel file name: Analysis truss network method) and the erythrocyte parameters recorded from the microscope images (Microsoft excel file name: Dataset for Erythrocytes). The Microsoft Excel spreadsheet was also used in processing the data to eliminate the effect of size from the dataset of the morphological parameters and also get the exclusive ranges of values for paired progenies. Tables included in this data article are processed data encompassing the exclusive ranges and percentages of erythrocytes of (i) pure *C. gariepinus* vs *P. hypophthalmus* (Tables 1); (ii) Clarias-like Clariothalmus vs pure *C. gariepinus* & Clarias-like Clariothalmus vs pure *P. hypophthalmus* (Tables 2);(iii) Panga-like Clariothalmus vs pure *C. gariepinus* & Panga-like Clariothalmus vs pure *P. hypophthalmus* (Tables 3); (iv) Pangapinus vs pure *C. gariepinus* & Pangapinus vs pure *P. hypophthalmus* (Tables 4); (v) Clarias-like Clariothalmus vs Panga-like Clariothalmus; Clarias-like Clariothalmus vs Pangapinus & Panga-like Clariothalmus vs Pangapinus (Tables 5). Also, the figure included in this data article shows for the very first time, different morphological abnormality of table-sized Clarias-like Clariothalmus hybrid (Fig. 1). While Fig. 2 showed the various erythrocyte data measured as presented in this study.

**Table 1:** Comparison of the exclusive ranges and percentages of the erythrocyte characteristics between *Pangasianodon hypophthalmus* and *Clarias gariepinus* (*n* = 500). Minimum – maximum exclusive range observed (percentage).

Parameter	Pure C. gariepinus vs Pure P. hypophthalmus	
	Pure ♀CG × ♂CG	Pure ♀pH × ♂PH
CMA (µm)	6.75 - 8.51 (49.4%)	10.80 - 14.15 (39.4%)
CMiA (µm)	9.14 - 9.27 (0.2%)	4.79 - 6.05 (0.8%)
NMA (µm)	2.97 - 3.10 (1.8%)	4.68 - 5.82 (9.8%)
NMiA (µm)	3.71 - 3.83 (1.4%)	2.13 - 2.75 (28.0%)
CA (µm^2^)	43.15 - 43.61 (0.2%)	91.41 - 106.58 (16.2%)
CV (µm^3^)	- (-)	431.48 - 488.57 (5.2%)
NA (µm^2^)	- (-)	7.91 - 8.81 (4.8%)
NV (µm^3^)	34.56 - 35.27 (0.2%)	9.18 - 13.03 (11.0%)

**Key:** CMA = Cell major axis; CMiA = Cell minor axis; NMA = Nuclear major axis; NMiA = Nuclear minor axis; CA = Cell area; CV = Cell Volume; NA = Nuclear Area; NV = Nuclear volume.

**Table 2:** Exclusive ranges and percentages of the erythrocyte characteristics of Clarias-like progenies from the ♀*Clarias gariepinus* × ♂*Pangasianodon hypophthalmus* cross in comparison with the pure parents (*n* = 500). Minimum – maximum recorded range (percentage).

Parameter	Exclusive range with ♀CG × ♂ CG		Exclusive range with ♀pH × ♂ PH	
	Pure ♀CG × ♂CG	Clarias-like ♀CG × ♂PH	Pure ♀pH × ♂PH	Clarias-like ♀CG × ♂PH
CMA (µm)	- (0.0%)	10.82 - 11.64 (3.0%)	11.65 - 14.15 (11.0%)	6.45 - 8.50 (13.8%)
CMiA (µm)	- (0.0%)	5.84 - 6.24 (1.2%)	4.79 - 5.71 (0.4%)	9.14 - 9.77 (5.8%)
NMA (µm)	4.59 - 4.67 (0.2%)	2.96 - 2.97 (0.2%)	4.69 - 5.82 (12.8%)	2.96 - 3.11 (3.6%)
NMiA (µm)	- (0.0%)	2.54 - 2.78 (2.2%)	2.13 - 2.53 (9.4%)	3.71 - 3.84 (1.6%)
CA (µm^2^)	- (0.0%)	91.47 - 101.54 (8.0%)	102.07 - 106.57 (0.6%)	37.66 - 43.56 (1.0%)
CV (µm^3^)	- (0.0%)	430.36 - 519.64 (7.4%)	109.49 - 115.27 (0.2%)	506.90 - 519.64 (0.6%)
NA (µm^2^)	17.74 - 16.47 (0.2%)	8.29 - 8.80 (1.8%)	16.55 - 18.93 (4.2%)	- (0.0%)
NV (µm^3^)	32.74 - 35.27 (0.4%)	11.14 - 13.05 (1.8%)	9.18 - 11.22 (3.2%)	- (0.0%)

**Key:** CMA = Cell major axis; CMiA = Cell minor axis; NMA = Nuclear major axis; NMiA = Nuclear minor axis; CA = Cell area; CV = Cell Volume; NA = Nuclear Area; NV = Nuclear volume.

**Table 3:** Exclusive ranges and percentages of the erythrocyte characteristics of Panga-like progenies from the ♀*Clarias gariepinus* × ♂*Pangasianodon hypophthalmus* cross in comparison with the pure parents (*n* = 500). Minimum – maximum recorded range (percentage).

Parameter	Exclusive range with ♀CG × ♂ CG		Exclusive range with ♀pH × ♂ PH	
	Pure ♀CG × ♂CG	Panga-like ♀CG × ♂PH	Pure ♀pH × ♂PH	Panga-like ♀CG × ♂PH
CMA (µm)	6.75 - 7.58 (3.0%)	10.83 - 12.82 (5.0%)	12.93 - 14.15 (1.0%)	7.60 - 8.51 (8.0%)
CMiA (µm)	6.28 - 6.71 (1.6%)	9.28 - 11.60 (12.8%)	4.79 - 6.71 (5.2%)	9.14 - 11.60 (17.8%)
NMA (µm)	2.97 - 3.03 (0.4%)	4.70 - 6.55 (7.6%)	- (0.0%)	5.95 - 6.55 (1.0%)
NMiA (µm)	- (0.0%)	3.83 - 5.47 (15.4%)	2.13 - 2.73 (26.0%)	3.71 - 5.47 (18.0%)
CA (µm^2^)	43.15 - 56.91 (10.2%)	91.57 - 146.78 (14.0%)	43.61 - 56.84 (0.6%)	107.91 - 146.78 (4.4%)
CV (µm^3^)	144.48 - 204.73 (6.8%)	430.65 - 880.32 (17.2%)	109.49 - 200.46 (0.8%)	497.32 - 880.32 (6.4%)
NA (µm^2^)	8.82 - 8.89 (0.6%)	17.78 - 33.42 (11.0%)	7.91 - 8.93 (4.8%)	19.00 - 33.42 (8.8%)
NV (µm^3^)	- (0.0%)	35.51 - 89.37 (12.4%)	9.18 - 12.82 (10.4%)	35.87 - 89.37 (11.8%)

**Key:** CMA = Cell major axis; CMiA = Cell minor axis; NMA = Nuclear major axis; NMiA = Nuclear minor axis; CA = Cell area; CV = Cell Volume; NA = Nuclear Area; NV = Nuclear volume.

**Table 4:** Exclusive ranges and percentages of the erythrocyte characteristics of the progenies from the ♀*Pangasianodon hypophthalmus* × ♂*Clarias gariepinus* cross in comparison with the pure parents (*n* = 500). Minimum – maximum exclusive range observed (percentage).

Parameter	Exclusive range with ♀CG × ♂ CG		Exclusive range with ♀pH × ♂ PH	
	Pure ♀CG × ♂CG	♀pH × ♂CG Progeny	Pure ♀pH × ♂PH	♀pH × ♂CG Progeny
CMA (µm)	6.75 - 8.28 (31.0%)	10.80 - 13.27 (38.4%)	13.27 - 14.15 (0.2%)	8.29 - 8.51 (0.2%)
CMiA (µm)	8.94 - 9.27 (1.4%)	5.74 - 6.28 (1.4%)	8.96 - 9.14 (0.8%)	- (0.0%)
NMA (µm)	2.97 - 3.03 (0.2%)	4.70 - 5.33 (3.8%)	5.45 - 5.82 (0.6%)	3.04 - 3.11 (0.4%)
NMiA (µm)	3.71 - 3.83 (1.4%)	2.12 - 2.75 (26.8%)	3.71 - 3.69 (0.2%)	2.11 - 2.13 (0.2%)
CA (µm^2^)	43.15 - 57.99 (13.2%)	91.43 - 98.75 (4.2%)	99.07 - 106.58 (3.2%)	- (-)
CV (µm^3^)	144.48 - 185.75 (1.8%)	454.12 - 429.00 (0.4%)	454.01 - 488.57 (1.8%)	- (-)
NA (µm^2^)	17.74 - 16.78 (0.2%)	7.59 - 8.77 (2.2%)	16.84 - 18.93 (2.4%)	7.59 - 7.85 (0.4%)
NV (µm^3^)	32.74 - 35.27 (0.4%)	8.42 - 13.04 (8.0%)	34.56 - 32.51 (0.2%)	8.41 - 9.18 (0.2%)

**Key:** CMA = Cell major axis; CMiA = Cell minor axis; NMA = Nuclear major axis; NMiA = Nuclear minor axis; CA = Cell area; CV = Cell Volume; NA = Nuclear Area; NV = Nuclear volume.

**Table 5:** Comparison of the exclusive ranges and percentages of the erythrocyte characteristics between progenies of the reciprocal crosses of *Pangasianodon hypophthalmus* and *Clarias gariepinus* (*n* = 500). Minimum – maximum exclusive range observed (percentage).

Parameter	Within Clariothalmus		Clariothalmus vs Pangapinus			
	Clarias-like ♀CG × ♂PH	Panga-like ♀CG × ♂PH	Clarias-like ♀CG × ♂PH	♀pH × ♂CG Progeny	Panga-like ♀CG × ♂PH	♀pH × ♂CG Progeny
CMA (µm)	6.45 - 7.58 (3.2%)	11.81 - 12.82 (0.6%)	6.45 - 8.28 (8.6%)	11.65 - 13.27 (8.6%)	7.60 - 8.29 (4.4%)	13.16 - 13.27 (0.6%)
CMiA (µm)	5.83 - 6.71 (4.2%)	9.81 - 11.60 (4.6%)	8.95 - 9.77 (8.2%)	5.74 - 5.84 (0.2%)	8.93 - 11.60 (26.4%)	5.74 - 6.72 (6.0%)
NMA (µm)	2.96 - 3.03 (0.8%)	4.63 - 6.55 (8.0%)	2.96 - 3.03 (0.8%)	4.60 - 5.33 (6.0%)	5.47 - 6.55 (3.0%)	3.03 - 3.04 (0.2%)
NMiA (µm)	2.54 - 2.73 (1.0%)	3.85 - 5.47 (15.0%)	3.70 - 3.84 (2.0%)	2.12 - 2.53 (7.2%)	3.69 - 5.47 (18.4%)	2.13 - 2.74 (25.8%)
CA (µm^2^)	37.66 - 56.76 (5.8%)	102.10 - 146.78 (5.0%)	37.66 - 56.75 (6.8%)	- (-)	98.90 - 146.78 (6.8%)	- (-)
CV (µm^3^)	115.28 - 204.53 (5.4%)	522.06 - 880.32 (5.2%)	455.11 - 519.64 (3.6%)	- (-)	454.64 - 880.32 (11.2%)	189.20 - 198.56 (0.8%)
NA (µm^2^)	8.29 - 8.92 (2.6%)	16.50 - 33.42 (14.0%)	- (-)	7.59 - 8.11 (0.8%)	16.87 - 33.42 (12.8%)	7.59 - 8.94 (2.8%)
NV (µm^3^)	11.13 - 12.78 (1.0%)	32.05 - 89.37 (15.8%)	- (-)	8.42 - 11.10 (2.2%)	32.60 - 89.37 (15.2%)	8.42 - 12.78 (6.6%)

**Key:** CMA = Cell major axis; CMiA = Cell minor axis; NMA = Nuclear major axis; NMiA = Nuclear minor axis; CA = Cell area; CV = Cell Volume; NA = Nuclear Area; NV = Nuclear volume.

## 2 Experimental design, materials and methods

Matured broodstocks of *P. hypophthalmus* (pH) and *C. gariepinus* (CG) (weighing between 1 – 2.5 kg) were reciprocally crossed to obtain novel hybrids (i.e. ♀CG × ♂pH aka Clariothalmus and ♀pH × ♂CG aka Pangapinus) following the methods used by Okomoda et al., [6] for the same crosses. The larvae of the different progenies were maintained until market size using commercial diets. Thereafter, morphological characterization through traditional measurement and truss network measurement of the progenies aged between four and six months was done.

Conventional traditional methods of determining morphometric measurement and meristic counts were done according to the method earlier described by Teugels et al. [2] and Solomon et al. [3]. The morphological variables taken included; Total length (TL), Standard length (SL), Dorsal fin length (DFL), Dorsal fin height (DFH), Distances between dorsal fin end and adipose fin origin (DBDAF), Pre dorsal distance (PDD), Pelvic fin length (PFL), Pelvic fin height (PFH), Pre pelvic distance (PPD), Pectoral fin length (PeFL), Pectoral fin height (PeFH), Anal fin length (AFL), Anal fin height (AFH), Caudal fin length (CFL), Caudal fin height (CFH), Caudal peduncle depth (CPD), Body height (BH), Body width (BW), Head length (HL), Head width (HW), Eye diameter (ED), Upper maxillary barbel length (UMBL), Lower maxillary barbel length (LMBL), Mouth width (MW), Pre-orbital length (POL). The meristic counts, however, were the numbers of dorsal fin ray, pelvic fin ray, pectoral fin ray, anal fin ray, and caudal fin ray

The collection of the truss network data of the morphological traits was in line with the methods of Turan et al. [4] with some modifications based on the peculiarities of the species been examined. Ten landmark point were identified and they are namely; Snout, origin of dorsal fin, posterior end of the dorsal fin, dorsal attachment of the caudal fin to the tail, ventral attachment of the caudal fin to the tail, posterior end of the anal fin, origin of the anal fin, origin of the pelvic fin, origin of the pectoral fin and the posterior point of the eye. Out of these landmark points, thirty-six measurement between the landmark points were recorded following Okomoda et al., [1].

To ensure that data variations can only be attributed to body shape differences and not size, the allometric formula given by Elliott et al. [7] was used to standardize the morphological data and eliminate size effect from the data set of the traditional morphometric measurement and truss network measurement.

M _adj_ = *M* (Ls / Lo) ^b^

Where *M* = original measurement,

M _adj_ = size-adjusted measurement,

Lo = TL of the fish,

Ls = Overall mean of the TL for all the progenies.

*b* = Estimated for each character from the collected data as the slope of the regression of log M on log Lo, using all fish of all the progenies.

However, meristic characters were not standardized as described above because they are independent of fish size [8, 9]. The percentages and exclusive ranges of the data gotten were also determined. This was done by first sorting the data in ascending order using the Microsoft Excel software. The sorted data of paired progenies were then compared to determine the range of values that are exclusive to each pair of the progenies examined. The paired combination evaluated includes; (i) pure *C. gariepinus* vs *P. hypophthalmus*; (ii) Clarias-like Clariothalmus vs pure *C. gariepinus*; (iii) Clarias-like Clariothalmus vs pure *P. hypophthalmus*; (iv) Panga-like Clariothalmus vs pure *C. gariepinus*; (v) Panga-like Clariothalmus vs pure *P. hypophthalmus*; (vi) Pangapinus vs pure *C. gariepinus*; (vii) Pangapinus vs pure *P. hypophthalmus*; (viii) Clarias-like Clariothalmus vs Panga-like Clariothalmus; (ix) Clarias-like Clariothalmus vs Pangapinus and (x) Panga-like Clariothalmus vs Pangapinus.

For the erythrocyte characterization, Ten fish each (*N* = 10) among the progenies of the different crosses and morphotypes were randomly selected for this characterization. Blood was collected from the caudal peduncle of the fish using an 18 gage needle fitted with a heparinized syringe. A dry blood smear was then prepared on a slide from the blood collected from each fish using the method previously specified by Felip et al., [10, 11], and Normala et al., [5]. The slide was observed under a Nikon Eclipse 80i compound microscope at 100 × magnification. The erythrocyte that was smeared on the slide was section and then five hundred (*n* = 500) erythrocytes were measured [5]. The parameters measured were cell major axis, cell minor axis, nucleus major axis, and nucleus minor axis. From these parameters, the cell area, nucleus area, cell volume, and nucleus volume, were calculated using the Microsoft Excel software following the formulae below.

Area of erythrocyte = *A* × *B*

Volume of erythrocyte = 4/3 × π × (A/2) × (B/2)^2^

Similar to what was done for morphological data, each parameter measured/calculated (i.e. axis, volume, and area) were sorted in ascending order using the Microsoft Excel software, and comparison was done for the data in pairs. The range of values that are exclusive to each pair of the progenies examined was determined, hence, the exclusive ranges and percentages of the paired combination of the different progenies were recorded.

## Ethics statement

The experimental protocols for this research were approved by the Universiti Malaysia Terengganu committee on research. All methods used involving the care and use of animals were following international, national, and institutional guidelines. This includes the U.K. Animals (Scientific Procedures) Act, 1986 and associated guidelines, EU Directive 2010/63/EU for animal experiments, as well as the National Institutes of Health guide for the care and use of Laboratory Animals (NIH Publications No. 8023, revised 1978)

## Declaration of Competing Interest

The authors declare that they have no known competing financial interests or personal relationships which have, or could be perceived to have, influenced the work reported in this article.

## Appendix Supplementary materials

Image, application 1

Image, application 2

Image, application 3

Image, application 4
